# Mesenchymal Stem Cells: What We Have Learned and How to Manage Them

**DOI:** 10.3390/biology14010001

**Published:** 2024-12-24

**Authors:** Nadia Lampiasi

**Affiliations:** Istituto per la Ricerca e l’Innovazione Biomedica, Consiglio Nazionale delle Ricerche, Via Ugo La Malfa 153, 90146 Palermo, Italy; nadia.lampiasi@irib.cnr.it; Tel.: +39-091-680-9513; Fax: +39-091-689-5548

Regenerative medicine is the new frontier of medicine. The possibility of replacing and/or repairing damaged tissues and organs represents a challenge to be overcome in the future. Organ transplantation is still a rare and risky practice. Instead, stem cell (SC) therapy has shown great promise in recent years [1,2]. SCs can be retrieved from different sites in the human body, but not all have the same differentiation potential. Mesenchymal stem cells (MSCs) are derived from the mesoderm and can differentiate towards adipogenic, osteogenic, chondrogenic, neurogenic and myogenic lineages [1,2]. MSCs are plentiful and can be sourced from bone marrow [3], adult peripheral blood [4], umbilical cord [5], adipose tissue [6] and dental pulp [7]. Their differentiation may depend on signals from the microenvironment and from the cells present in the niche, all factors that can induce cellular migration, proliferation and differentiation. The use of MSCs for repair and/or regeneration has proven challenging, as MSCs struggle to target the damaged site [8]; have a short half-life [9]; has great variability [10]; and may pose a risk to human health, as they can degenerate.

An alternative to stem cell transplantation is cell homing. This method is based on the recruitment and migration of endogenous MSCs and is a more effective and safer strategy than cell transplantation. For a long-lasting and effective therapy based on the use of endogenous MSCs, the two challenges to address are cell migration and aging. One review and one article in this Special Issue address these issues. Human dental pulp stem cells (hDPSCs) are MSCs obtained from dental pulp and derived from neural crests. They can differentiate into odontoblasts, osteoblasts, chondrocytes, adipocytes and nerve cells based on signals received from the microenvironment. DPSCs use modalities known as the mesenchyme and the amoeboid for migration, which in response to biochemical and biophysical signals from the microenvironment, produce lamellipodia, filopodia, blebs and mygrasomes. In this review [11], the most recent works on the intriguing role that cell migration could have on the fate of DPSCs are presented, suggesting that different methods to obtain DPSCs (explant and digestion) produce SCs with different potentialities (Figure 1).

MSCs have a limited number of proliferation cycles and are subject to the phenomenon of aging, which represents a limiting factor for their use in therapy. This limitation is mainly due to the low activity of endogenous telomerase. To solve this problem, several methods have been used, not without risks for human health. Viral genes such as human papillomavirus (HPV) [12], simian virus 40 (SV40) [13], or human telomerase gene transfer [14] or oncogenes [15] can be used. In particular, introducing the human telomerase reverse transcriptase (TERT) gene allows us to provide a safer and more suitable solution. It involves improving the activity of endogenous telomerase and prolonging the cell lifespan, thus increasing the number of cellular passages [16]. In this study, MSCs are isolated from sheep umbilical cords (UCMSCs) and then are immortalized by transfecting the human TERT gene [17]. This approach was effective in inhibiting senescence and promoting cell proliferation compared to normal UCMSCs. Furthermore, immortalized UCMSCs were not tumorigenic and maintained stem cell characteristics, suggesting great potential in medical applications (Figure 2).

Another alternative to MSC transplantation is the use of MSC-free components such as the secretome, the cell extract (CE), and the conditioned culture medium (CM) coming from the MSCs, which are systems considered more suitable for human therapy because they are safer and therefore more effective [18,19,20,21,22] (Figure 3).

The cell extract (CE) is the heterogeneous mixture isolated from soluble components of cell lysates. It contains proteins, nucleic acids, lipids, carbohydrates, and organelles from cells. Recently, many interesting manuscripts have been published on this topic, including a review in this Special issue [22]. In this review, the authors compare the effects of CE from MSCs with those from other cells/tissues and evaluate the potential in mitigating the damage of irradiated salivary glands in head and neck cancer patients, suggesting the benefits of using CE from MSCs. On the other hand, just to mention some of the most recent works, it has been demonstrated that CE from adipose tissue-derived MSC spheroids suppress lymphocyte activation while enhancing T regulatory cell expansion [23], the CE from MSCs stimulate the migration and proliferation of periodontal ligament (PDL) cells [24] and mitochondria from bone marrow-derived MSCs has emerged as a promising therapy for recovery from central nerve injury [25].

The secretome is the set of extracellular vesicles (EV) and exosomes (EX) produced by MSCs. It contains miRNAs, mRNAs, proteins, cytokines and other biological signals with high functional value. The secretome is widely studied for its high potential in promoting tissue regeneration. Among the manuscripts investigating this topic, the authors of [26] analyzed miRNA expressions to study the priming effects on intracellular MSC-derived and EX-derived miRNAs. IFN-γ treatment and 3D-MSC cultivation techniques, used as priming, demonstrated that all miRNAs produced are associated with tissue repair/regeneration (Figure 4).

Many systematic reviews have been performed to study the various effects of the secretome in tissue/organ rejuvenation/regeneration. This systematic review aims to assess the effects of MSC-EVs on follicle number, hormonal profile and fertility in primary ovarian insufficiency (POI) animal models, suggesting a beneficial effect since MSC-EV treatment significantly increased total follicle number [27]. Another systematic review investigated MSC-EVs on liver fibrosis, suggesting significantly improved liver function with the promotion of damaged liver tissue repair, and progression-slowing effects of hepatic fibrosis [28]. On the other hand, recent research has highlighted the effects of MSC-EVs as a potential therapeutic approach for anti-tumor strategies. Among others, a recent review identified several key studies demonstrating the anti-tumor effects of MSC-EVs in glioblastoma (GBM) models [29].

As mentioned above, senescence is a limitation to the use of MSCs in regenerative medicine, and it is also a limitation to the use of its secretome. In fact, it has been shown that the secretome from senescent MSCs is different from that of younger SCs and also has a reduced potential to promote cellular differentiation [30]. On the other hand, the secretome from young MSCs can slow down the process of MSC senescence [31]. The goal of the research is therefore to find a way to keep MSCs or their derivatives always young, so as to always have a means available to treat cellular/tissue damage.

**Conclusions**

The final considerations that we can deduce are that MSCs and/or their derivatives certainly represent a turning point in medicine 4.0, even if there are still several points to be investigated carefully for a safe and long-lasting therapeutic approach. However, given the large number of studies carried out in recent years, it is plausible that there will soon be an answer to these questions that are still unclear. The hope is that the studies will proceed quickly and that researchers will clarify the fundamental points for the safe use of MSCs in regenerative medicine.

## Figures and Tables

**Figure 1:**
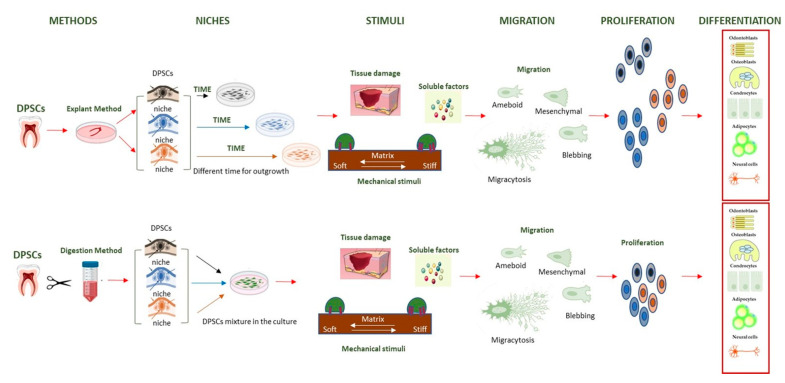
Schematic representation of the two methods to obtain DPSCs from teeth. Adopted from [11].

**Figure 2:**
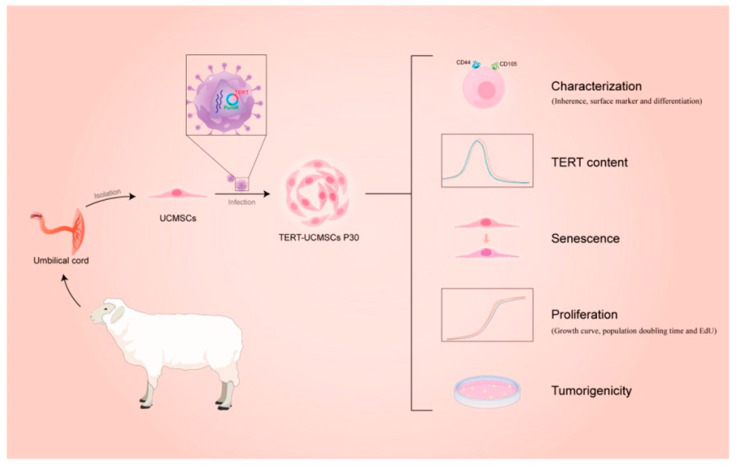
Schematic representation of the experimental method followed in this research. Adopted from [17].

**Figure 3:**
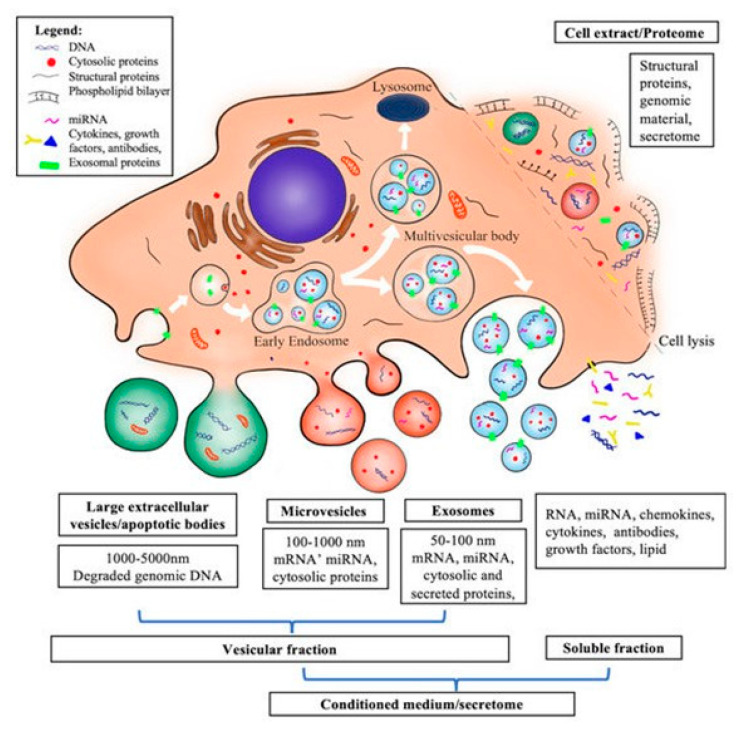
Schematic representation of the obtained cell-free components of MSCs. Adopted from [22].

**Figure 4:**
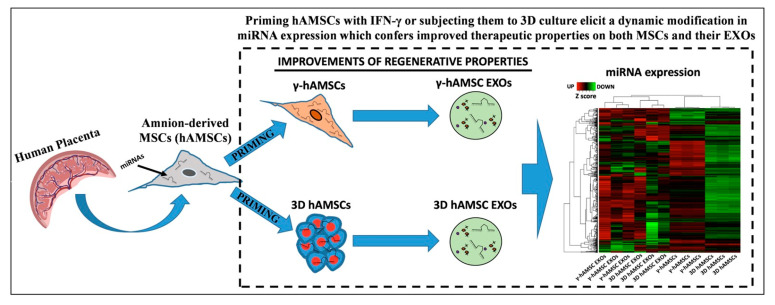
Schematic representation of the experimental method followed in this research. Adopted from [26].
